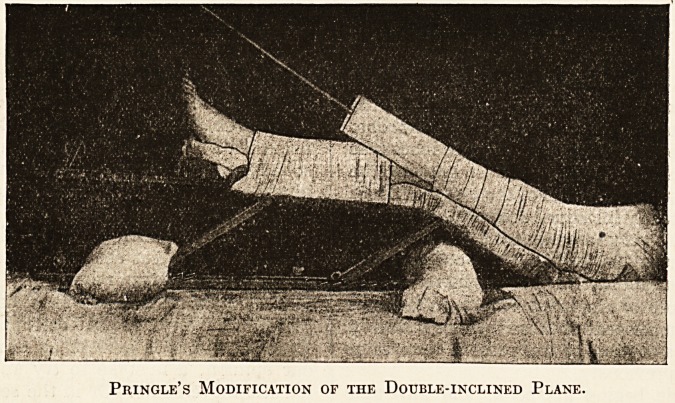# The Modern Treatment of Fractures
**Fractures and their Treatment*, by J. Hogarth Pringle, F.R.C.S. London: Henry Frowde, and Hodder and Stoughton, "Oxford Medical Publications," 1910. 12s. 6d. net.


**Published:** 1910-10-15

**Authors:** 


### SPECIAL ARTICLES.

### THE MODERN TREATMENT OF FRACTURES.*

"Paradoxical as the statement may appear, the subject of fractures is a comparatively new one. This is to a large extent the result of the insurance of workmen against injuries, and in part to the advent of Röntgen's discoveries of the *x*-rays. Both factors led to a greatly increased activity in the study of fractures, and a very large amount of work, both clinical and experimental, has been put out in consequence; the after-effects have been more closely studied; new types of fractures have been brought to light, and the treatment of these injuries probably receives greater attention at the present time than it has ever done previously."

So Mr. Pringle begins his book, and although he modestly calls it a compilation, we feel when we lay it down that it is a highly useful practical work, containing an amount of concentrated information that is almost startling by reason of its importance. It is the first English monograph that deals thoroughly with fractures, and although *précis* and condensation are necessary when we consider that the author has only some 360 odd pages of large, easy print available, it is surprising to note how well the process of boiling down has been managed. For the rest, it is only necessary to say that the book comes from the publishers of the Oxford Medical Manuals, and is included in their Medical Publications series; these are credentials which should ensure it a welcome anywhere among practitioners. And this is essentially a practitioner's work. The hospital surgeon will find comparatively little that is strikingly new here, although even for one who daily dabbles in plaster and in skiagraphic examinations it is interesting. The first part deals with general considerations, and is wholly admirable—even when, meticulously, one deplores the brief mention of bone cysts as a cause of fracture at the expense of a somewhat longer note on aneurism of the aorta as a causative factor. Personally we should have thought that it was far more important to deal with cysts, which are not so rare as is supposed, which may cause strange and weird fracture phenomena, and the diagnosis of which means a very great deal to the patient. Again, to be meticulous once more, why leave out in the lengthy note on crepitus as a sign of fracture the sometimes very useful osteophonic percussion? Unless crepitus is evident on examination it is, in our opinion, a mistake to try and elicit it by systematic movements. Or in the section dealing with causes of non-union, why omit tight splinting and absolute immobility? There have been cases in which the surgeon wired the bones so tightly that union did not result until the joint was loosened. It is well to draw attention to the importance of helping nature along by a little judicious worrying! But these cavillings are side matters, and the main point after all is treatment.

We note with pleasure Mr. Pringle's soundness and moderation, both of which are in agreement with the consensus of surgical opinion. To wire or not to wire is far too commonly the question in cases where conservative methods should only be considered. Here the reader will find a full *résumé* of all the available methods, from the old splint method to Lucas-Championniere's early mobilisation splintless method, which is nowadays so often and so successfully tried in cases of Colles' fracture. The author does not approve of immediate plaster immobilisation; in our opinion he lays too much stress on the objections to this method, since a well-applied Croft, for example in a case of Pott's fracture, does excellent service, and by equal, and to some extent elastic, pressure really minimises the amount of swelling. The *sine qua non*, of course, is that such

* *Fractures and their Treatment*, by J. Hogarth Pringle, F.R.C.S. London: Henry Frowde, and Hodder and Stoughton, "Oxford Medical Publications," 1910. 12s. 6d. net.

plaster splints should be very carefully applied, preferably only when the patient is fully anæsthetised. No mention is made of the much lighter and very excellent celluloid splints; they are, it is true, rarely used in English practice, since most of us do not know the multifarious uses of the acetone pot, but they are well worthy of a trial. Deserved stress, on the other hand, is laid on the importance of extension, and this part of the work demands the practitioner's careful attention. The diagrams showing longitudinal and lateral traction are good, and the descriptive letterpress is quite clear, so that this part is really very helpful. The short discussion on open methods is impartial, and puts the case very well. We note with some surprise that the author favours fixing by wire in all cases of compound fracture; these are really the most difficult class of cases to keep aseptic, and, in general practice at least, preferably treated by conservative methods. The ambulant treatment is not so fairly presented; in Mr. Pringle's opinion it is only suitable in a few extraordinary cases. With proper apparatus, fixed under the surgeon's own supervision and not left to the care of instrument-makers, the ambulant method is, in our opinion, a good one, and certainly, from the patient's point of view, to be preferred to splinting. In the case of children, for example, the ambulant method for fractures of the lower humeral shaft, by means of a weight carried in the patient's hand, is certainly an effective method, and ought to have been dealt with.

### Special Fractures.

Coming to special fractures, the book contains much that will be interesting and novel to those who have not followed the newer Continental methods, especially those of the Cologne school. Fractures of the skull, trachea, vertebræ, and pelvis are well described. Unfortunately the treatment here is scarcely modern, for we have not advanced much since the days when Hippocrates proposed to treat a fracture dislocation of the spine by opening the abdomen and reducing the fracture by digital pressure! In the treatment of pelvic fractures, somewhat more should be stated about the desirability of fixation in plaster if necessary; the obstinate sciatica that not infrequently results from ordinarily treated cases the general practitioner has to cope with, not the hospital surgeon. The author describes his method of treating fractures of the former by his adjustable plane (a photograph of which, by courtesy of the publishers, we are enabled to attach herewith). This seems a good modification of the usual method. The angles of the splint can be varied to suit any particular case, and an extension cord, acting in the leg axis over two distal pulleys, is a useful adjunct. This method seems specially suitable for shaft fractures just below the trochanter, and does not press upon the posterior vessels and nerves so much as the middle angle of the ordinary double-inclined plane does. Hodgen's and Bryant's methods are well described, while the Long Liston (not, however, Desault's) splint is illustrated as well. We are particularly struck with the excellence of the illustrations and the descriptions of apparatus in the book. The photographs showing the examination for fractures at the lower end of the leg, the various modifications of clavicular fracture bandages, and the application of Middeldorpf's splint in fractures of the arm bones are especially good. There is an admirably clear chapter on fractures and their results in connection with compensation claims; this is one of the most helpful articles on the subject that have appeared in print, and for it alone the practitioner should give the book a place in his library. Finally, there is a valuable bibliography, British as well as foreign, at the end, and a useful index.

Pringle's Modification of the Double-inclined Plane.

We have no space to deal in detail with the special fractures and their treatment. Every one is lucidly described, often with skiagraphic reproductions, which are much more useful than the diagrams one meets with in ordinary text-books. In view of the importance of the whole subject, especially in private practice, the book is a very serviceable one, which should attain wide popularity. It is thoroughly practical, well written, and contains many hints such as only a large and wide experience can give, and which are likely to prove of great help to those who have cases of fracture to treat.

## Figures and Tables

**Figure f1:**